# Downregulation of the psychiatric susceptibility gene *Cacna1c* promotes mitochondrial resilience to oxidative stress in neuronal cells

**DOI:** 10.1038/s41420-018-0061-6

**Published:** 2018-05-10

**Authors:** Susanne Michels, Goutham K. Ganjam, Helena Martins, Gerhard M. Schratt, Markus Wöhr, Rainer K. W. Schwarting, Carsten Culmsee

**Affiliations:** 1 Institute of Pharmacology and Clinical Pharmacy, Philipps-University, Marburg, Germany; 2 Center for Mind, Brain and Behavior, Philipps-University, Marburg, Germany; 3 Department of Health Sciences and Technology, Systems Neuroscience, ETH Zurich, Zurich, Switzerland; 4 Department of Experimental and Biological Psychology, Behavioral Neuroscience, Philipps-University, Marburg, Germany

## Abstract

Affective disorders such as major depression and bipolar disorder are among the most prevalent forms of mental illness and their etiologies involve complex interactions between genetic and environmental risk factors. Over the past ten years, several genome wide association studies (GWAS) have identified *CACNA1C* as one of the strongest genetic risk factors for the development of affective disorders. However, its role in disease pathogenesis is still largely unknown. Vulnerability to affective disorders also involves diverse environmental risk factors such as perinatal insults, childhood maltreatment, and other adverse pathophysiological or psychosocial life events. At the cellular level, such environmental influences may activate oxidative stress pathways, thereby altering neuronal plasticity and function. Mitochondria are the key organelles of energy metabolism and, further, highly important for the adaptation to oxidative stress. Accordingly, multiple lines of evidence including post-mortem brain and neuro-imaging studies suggest that psychiatric disorders are accompanied by mitochondrial dysfunction. In this study, we investigated the effects of *Cacna1c* downregulation in combination with glutamate-induced oxidative stress on mitochondrial function, Ca^2+^ homeostasis, and cell viability in mouse hippocampal HT22 cells. We found that the siRNA-mediated knockdown of *Cacna1c* preserved mitochondrial morphology, mitochondrial membrane potential, and ATP levels after glutamate treatment. Further, *Cacna1c* silencing inhibited excessive mitochondrial reactive oxygen species formation and calcium influx, and protected the HT22 cells from oxidative cell death. Overall, our findings suggest that the GWAS-confirmed psychiatric risk gene *CACNA1C* plays a major role in oxidative stress pathways with particular impact on mitochondrial integrity and function.

## Introduction

Affective disorders such as major depression (MDD) and bipolar disorder (BD) are among the most prevalent forms of mental illness and affect more than 350 million people worldwide^1^. Their etiologies involve complex interactions between genetic and environmental risk factors^2,3^. The pronounced heritability of affective disorders suggests that genetic influences play a key role in disease pathogenesis^4^. In fact, the heritability ranges from 40–50% in MDD to 80–85% in BD, and family studies indicate for MDD a two- to threefold and for BD a nearly tenfold increase in lifetime risk among first-degree relatives^5,6^. During the past decade, several genome wide association studies (GWAS) have identified *CACNA1C* as one of the strongest genetic risk factors for the development of affective disorders^7–9^. It has been shown that the main single nucleotide polymorphism (SNP) rs1006737 is associated with increased mRNA expression of *CACNA1C* and enhanced calcium signaling^10–12^. *CACNA1C* codes for the pore-forming α_1C_ subunit of Ca_V_1.2, the major L-type voltage-gated calcium channel in the brain. Brain Ca_V_1.2 channels couple Ca^2+^ influx to neuronal gene transcription and play an important role in synaptic plasticity, dendritic development, and cell survival^13,14^. However, the underlying cellular and molecular mechanisms explaining how this genetic risk variant contributes to the pathophysiology of neuropsychiatric disorders remain largely unknown^15,16^.

Vulnerability to affective disorders is not only based on genetic predisposition, but also involves diverse environmental risk factors such as intrauterine infections of the embryo, perinatal brain injuries attributed to birth complications, childhood maltreatment, migration, and other adverse pathophysiological and psychosocial life events^17–19^. There is accumulating evidence that such psychosocial stressors activate oxidative stress pathways, thereby disrupting the oxidant-antioxidant balance within the brain and causing oxidative damage to DNA, mitochondria, lipids, and proteins. Mitochondrial impairment, in turn, can accelerate the production of reactive oxygen species (ROS), which further modifies biological macromolecules and alters cellular functions^20^. In the brain, the accumulation of oxidative damage may result in loss of neuronal plasticity and function, and ultimately in apoptotic cell death, processes that have also been implicated as underlying mechanisms in the neuropathology of affective disorders^21,22^.

Mitochondria are the key organelles of energy metabolism and, thus, are highly important for adaptation to oxidative stress, calcium homeostasis, and regulation of cellular viability and function. Accordingly, numerous findings from neuro-imaging, post-mortem brain analyses, GWAS, and studies on peripheral biomarkers suggest that psychiatric disorders are accompanied by mitochondrial dysfunction^23,24^. These investigations revealed reduced complex I activity, increased ROS levels, decreased ATP production, downregulated expression of anti-apoptotic proteins, and upregulated expression of pro-apoptotic proteins in patients suffering from affective disorders or schizophrenia, all indicating impaired mitochondrial function.

In the present study, neuronal HT22 cells were used to elucidate whether the psychiatric risk gene *Cacna1c* affects oxidative stress-mediated disruption of mitochondrial function and, thereby, neuronal cell homeostasis. The applied mouse hippocampal HT22 cell line allows for the targeted manipulation of gene expression and serves as a well-established model system for glutamate-induced oxidative stress with significant mitochondrial impairment^25,26^. The specific aim of this study was to investigate the effects of reduced *Cacna1c* gene expression in combination with oxidative stress on mitochondrial function, Ca^2+^ homeostasis, and cell viability.

## Results

### Downregulation of *Cacna1c* preserved mitochondrial morphology and function independent of glutathione depletion in glutamate-challenged HT22 cells

**Fig. 1:**
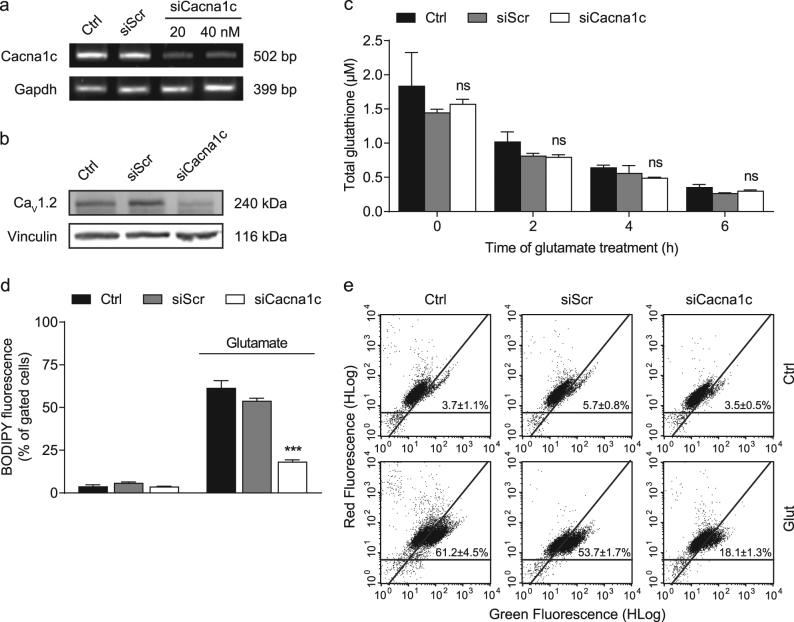
siRNA-mediated knockdown of *Cacna1c* prevented lipid peroxidation, but not glutathione depletion following glutamate exposure. **a** *Cacna1c* mRNA levels were analyzed 24 h after siRNA transfection with 20 and 40 nM. *Gapdh* served as internal control. **b** Protein samples were collected 48 h after transfection with 40 nM siRNA and the Ca_V_1.2 expression levels were then identified by Western blot. Vinculin was used as loading control. **c** Total glutathione levels were calculated from three replicates per condition after 0, 2, 4, and 6 h of glutamate treatment (10 mM). Data are provided as mean + SD. **d**, **e** Lipid peroxidation in HT22 cells was determined using BODIPY staining after an 8-h incubation with 9 mM glutamate. The dot plots show representative replicates and the bar graph summarizes the associated experiment where three replicates per sample are shown as percentage of cells in the upper right quarter (mean + SD; 10,000 cells per replicate). Ctrl control, siScr scrambled siRNA, siCacna1c *Cacna1c* siRNA, Glut glutamate. ***p < 0.001; ns (not significant) compared to glutamate-treated ctrl (ANOVA, Scheffé’s-test)

In order to study the effects of *Cacna1c* depletion on mitochondrial parameters, we first validated the siRNA-mediated knockdown at the level of mRNA and protein. As shown in Fig. 1a, *Cacna1c* siRNA significantly reduced mRNA expression by 75% compared to control and siScr. In line with this finding, the immunoblot revealed a pronounced downregulation of Ca_V_1.2 protein levels in siRNA-transfected HT22 cells (Fig. 1b). A comparable knockdown was also achieved using another *Cacna1c* siRNA sequence (siCacna1c 3; Supplementary Fig. S1).

To investigate the effects of *Cacna1c* gene silencing on the mechanisms of glutamate-induced oxidative stress, we next analyzed glutathione levels (GSH) and lipid peroxidation in HT22 cells exposed to glutamate. Total GSH were determined 2, 4, and 6 h after the onset of glutamate treatment (Fig. 1c). Loss of GSH marks the initial step in oxidative glutamate toxicity and in fact, we observed a steady decline in GSH concentration over time^27^. Notably, there were no significant differences in GSH depletion between *Cacna1c* siRNA-transfected HT22 cells and controls. Glutathione depletion leads to the accumulation of lipid peroxides, which was evaluated here 8 h post-glutamate treatment using the fluorescent dye BODIPY followed by flow cytometric analysis^26^. The results revealed that the initial rise in lipid peroxidation upon glutamate exposure was largely prevented in HT22 cells transfected with *Cacna1c* siRNA, indicating a partial action upstream of mitochondria in the considered oxidative stress cascade (Fig. 1d,e).

**Fig. 2:**
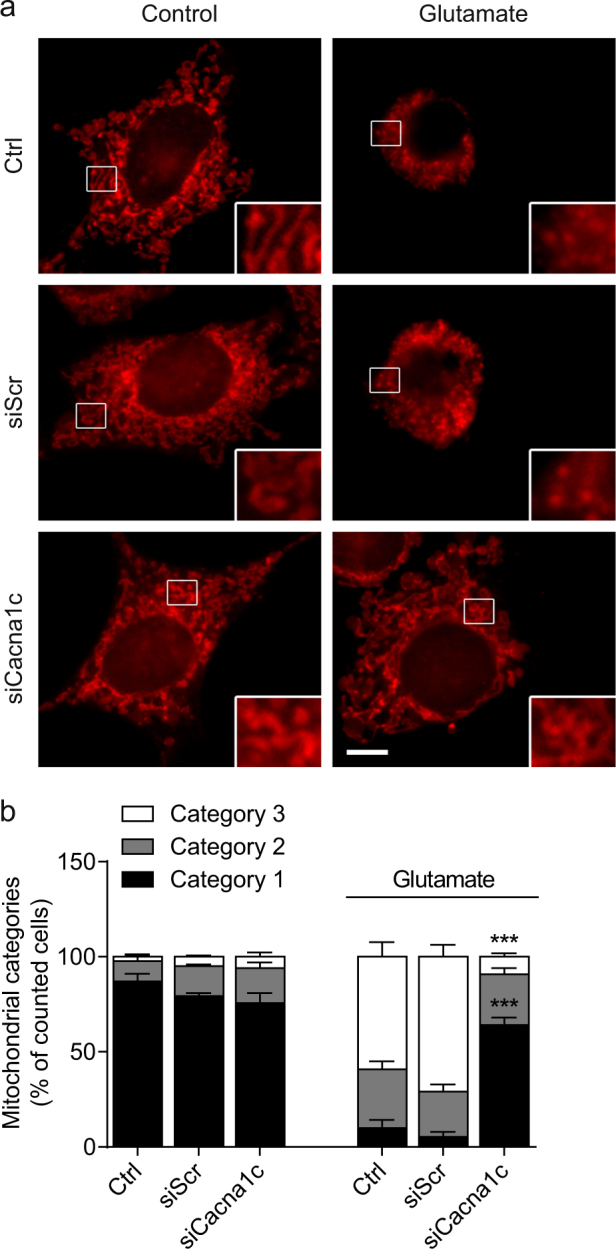
*Cacna1c* gene silencing preserved the morphology of healthy mitochondria in glutamate-challenged HT22 cells. **a** Changes in mitochondrial morphology were visualized after 15 h of glutamate exposure using MitoTracker Deep Red. Epifluorescence images of representative HT22 cells are shown with details enlarged by 300% at the bottom right. Scale bar 10 µm. **b** Five hundred cells per condition were classified into three categories of mitochondrial fragmentation (Category 1: elongated; Category 2: intermediate; Category 3: fragmented) and quantified as percentage of counted cells (*n* = 3, mean + SD). ***p < 0.001 compared to glutamate-treated ctrl (ANOVA, Scheffé’s-test)

Then, we focused on specific parameters of mitochondria, since these organelles play a crucial role in the employed paradigm of oxidative stress^28^. As displayed in Fig. 2a, we found a pronounced increase in mitochondrial fragmentation in the control and siScr-transfected cells after glutamate treatment. In contrast, *Cacna1c* siRNA-transfected cells mostly maintained their tubular, elongated morphology despite the glutamate challenge. The mitochondrial morphology was quantified by means of a classification into three different categories^25^. Briefly, cells containing healthy mitochondria with an elongated, tubule-like structure were defined as Category 1; while dying cells show strongly fragmented mitochondria accumulating around the nucleus and were classified as Category 3. Based on this classification system, the systematic quantification confirmed that *Cacna1c* gene silencing preserved mitochondrial morphology at Category 1 also after glutamate exposure (Fig. 2b). To substantiate these results, we additionally evaluated the expression of mitochondrial fission and fusion proteins, as well as mitochondrial biogenesis and mitophagy markers (Supplementary Fig. S2). While the total levels of mitofusin 2 (Mfn2), a protein that regulates the fusion of the outer mitochondrial membrane, were not significantly altered, we observed changes in the expression of the mitochondrial fission proteins Drp1 and Fis1. During mitochondrial fission, cytosolic dynamin-related protein 1 (Drp1) is recruited to the mitochondrial outer membrane where it interacts with mitochondrial fission protein 1 (Fis1) leading to constriction and fragmentation of the organelles. Upon glutamate challenge, the levels of both Drp1 and Fis1 were increased in the control conditions, whereas in the siCacna1c-transfected cells their levels remained unchanged. In addition to the rather moderate differences in the actual levels of the related fission and fusion proteins, it is likely that mitochondrial fragmentation is also regulated by modifications in their activity. The peroxisome proliferator-activated receptor gamma coactivator 1 alpha (PGC1α) is a transcription factor that regulates genes involved in mitochondrial biogenesis. In glutamate-treated HT22 cells, *Cacna1c* knockdown led to elevated PGC1α levels, contrary to controls showing slightly decreased levels. Sequestosome 1 (p62) is involved in the recruitment of autophagosomal membranes to the mitochondria and interacts with the microtubule-associated protein light chain 3 isoform B (LC3B). During autophagy LC3B-I (16 kDa) is converted to LC3B-II (14 kDa) through lipidation. The expression of p62 and LC3B-II was increased after 16 h of glutamate treatment in *Cacna1c* siRNA-transfected and control cells alike. However, silencing of Cacna1c induced already a considerable increase in basal levels of both p62 and LC3B-II compared to controls. This finding is consistent with earlier studies stating that decreases in cytosolic Ca^2+^ can induce autophagy^29^. Our results indicate that *Cacna1c* downregulation stimulates mitochondrial biogenesis under oxidative stress without affecting mitophagic processes, thereby promoting the turnover of mitochondria and preventing the accumulation of dysfunctional mitochondria in HT22 cells.

**Fig. 3:**
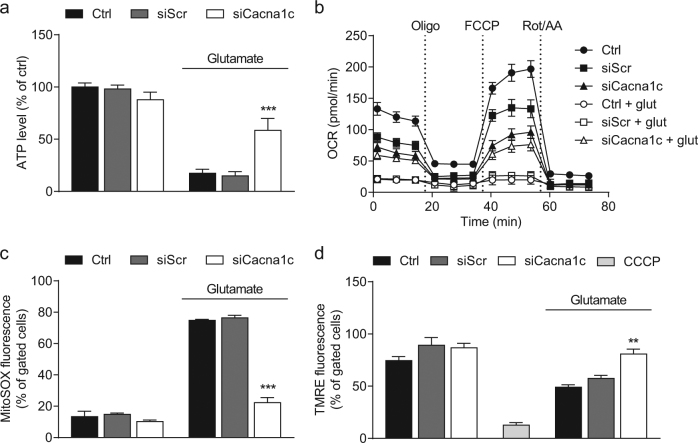
Downregulation of *Cacna1c* gene expression mediated protection of mitochondrial function against glutamate-induced oxidative stress. **a** A luciferase-based assay was used to determine ATP levels in glutamate-exposed (8 mM) HT22 cells. Values from eight replicate wells per condition are displayed as mean + SD. The luminescence under control conditions is set as 100%. **b** After 16 h of glutamate challenge, the oxygen consumption rate (OCR) was analyzed with a Seahorse XF^e^96 Analyzer. Data of 3–7 replicate wells per condition are given as mean ± SD. Oligo oligomycin, FCCP carbonyl cyanide 4-(trifluoromethoxy)phenylhydrazone, Rot rotenone, AA antimycin A. **c** Mitochondrial superoxide formation was investigated by flow cytometry using the fluorescent dye MitoSOX. HT22 cells were treated with 9 mM glutamate for 18 h. **d** Flow cytometric analysis of the mitochondrial membrane potential was realized via TMRE staining after 18 h of glutamate treatment. CCCP (carbonyl cyanide 3-chlorophenylhydrazone, 50 µM) is a mitochondrial membrane depolarizer and serves as positive control. **c**, **d** Each bar chart depicts one representative experiment with three replicates per sample (mean + SD; 10,000 cells per replicate). ***p < 0.001; **p < 0.01 compared to glutamate-treated ctrl (ANOVA, Scheffé’s-test)

At the functional level, we next determined the concentration of cellular adenosine triphosphate (ATP), which provides the energetic basis for neuronal plasticity, viability, and function. In line with the previous findings on mitochondrial morphology, the downregulation of *Cacna1c* blocked the loss of ATP levels in response to glutamate (Fig. 3a). To further confirm the protective effects of *Cacna1c* knockdown in this model of oxytosis, the oxygen consumption rate (OCR) was analyzed as a measure of mitochondrial respiration. As illustrated by the OCR graphs shown in Fig. 3b, the basal respiration, represented by the OCR before injection of oligomycin (Oligo), was considerably higher in *Cacna1c* siRNA-transfected cells compared to controls after glutamate treatment. In line with this observation, the maximal mitochondrial respiration, detected after FCCP injection, was also significantly higher in glutamate-exposed cells with *Cacna1c* depletion versus controls. Here, the mitochondrial uncoupler FCCP mimics an increased energy demand as, for example, present in times of stress and, thereby, allows for the evaluation of the respiratory reserve. Our findings imply that mitochondrial dysfunction attributed to oxidative glutamate toxicity leads to decreased stress adaptation, which is prevented by a knockdown of *Cacna1c*. Besides the effects of *Cacna1c* downregulation after glutamate challenge, differences in the OCR between untreated conditions and *Cacna1c* siRNA-transfected cells can be observed, which may be functionally relevant. Compared to untreated controls, silencing of *Cacna1c* led to a lower basal and maximal OCR without a compensatory increase in glycolysis, as measured by the extracellular acidification rate (ECAR; Supplementary Fig. 3a). Thus, consistent with the slightly decreased basal ATP levels, depletion of *Cacna1c* resulted in a reduced metabolic potential, suggesting in particular a constitutively inhibited respiratory chain (Fig. 3a and Supplementary Fig. 3b). Reportedly, the activity of the electron transport complex IV is disinhibited by increases in cytosolic calcium concentration ([Ca^2+^]_i_) and is thereby presumably dependent on *Cacna1c* function. Consequently, *Cacna1c* knockdown may act as a form of preconditioning that prevents glutamate toxicity via complex IV inhibition and concurrent decrease in mitochondrial ROS production^30^. As described previously, the mitochondrial electron transport chain is the major source of ROS in this paradigm of glutamate-induced stress. Therefore, mitochondrial superoxide levels were detected by MitoSOX Red staining^27^. Fig. 3c demonstrates that mitochondrial ROS formation was significantly elevated in glutamate-challenged HT22 cells, but not in the *Cacna1c* siRNA-transfected cells. This protective effect was confirmed by another siRNA sequence (siCacna1c 3; comparable 62% reduction of Ca_V_1.2 protein levels versus control), whereas a further siRNA (siCacna1c 2; only 21% reduction of Ca_V_1.2 protein levels compared to controls) did not provide sufficient knockdown to affect mitochondrial ROS levels in glutamate-treated cells (Supplementary Fig. S4a). These results suggest that the functional effects of *Cacna1c* siRNA transfection strongly correlate with the extent of reduction in mRNA and protein levels (Supplementary Fig. S1). Another common feature of apoptotic and metabolically stressed cells is the collapse of the mitochondrial membrane potential (ΔΨ_m_)^31^. According to Fig. 3d, the breakdown of ΔΨ_m_ in response to glutamate is indicated by a loss in TMRE fluorescence and this was visibly attenuated in *Cacna1c*-silenced cells. Furthermore, *Cacna1c* siRNA-transfected HT22 cells exhibited significantly less ΔѰ_m_ hyperpolarization in the surviving cell population after glutamate damage (Supplementary Fig. S5). A lower ΔѰ_m_ results from a decreased [Ca^2+^]_i_ and is accompanied by less mitochondrial superoxide formation (Fig. 3c)^32^. These results further substantiate our findings from the OCR measurements and assist in elucidating the protective mechanisms of *Cacna1c* downregulation.

**Fig. 4:**
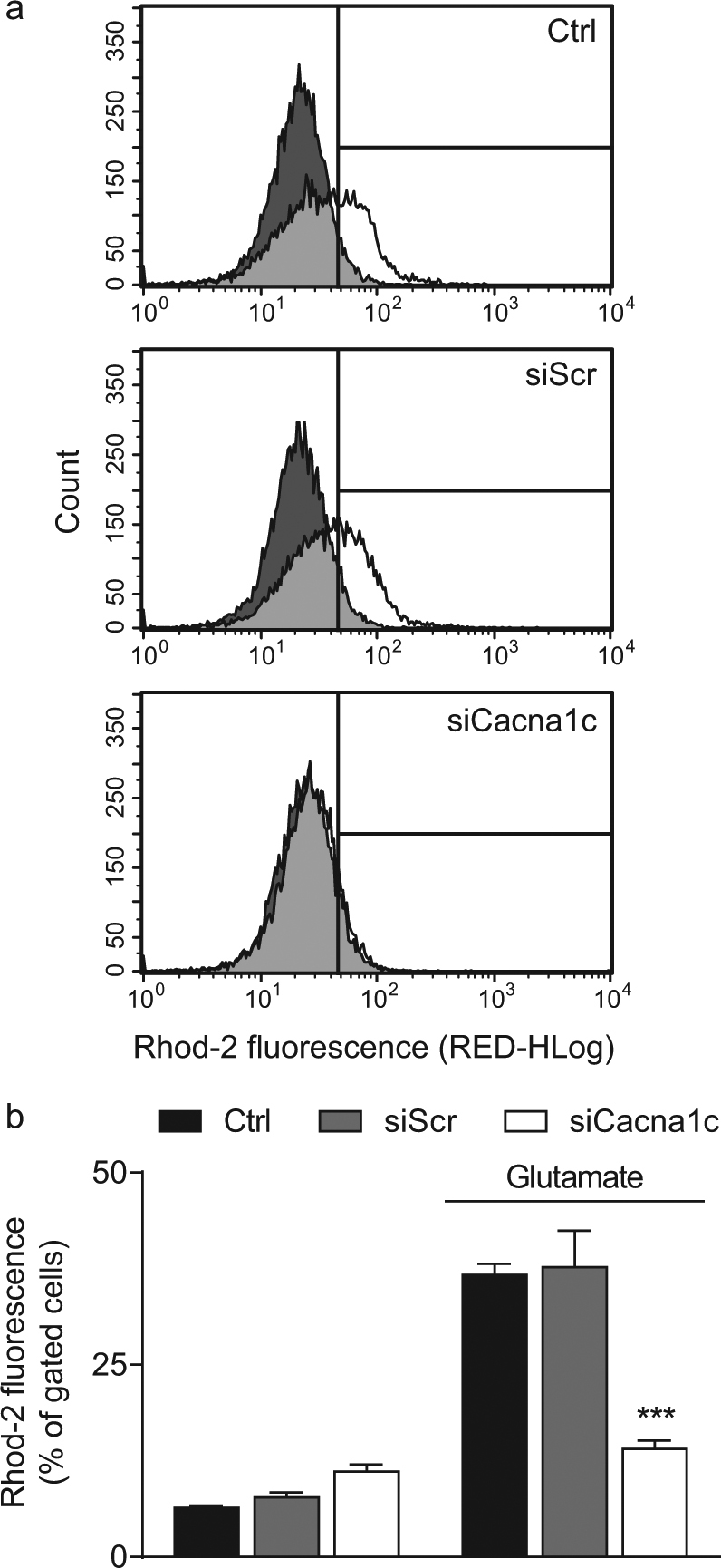
Depletion of *Cacna1c* attenuated the glutamate-induced rise in mitochondrial calcium concentration. **a** The mitochondria-specific dye dihydrorhod-2 AM was used for the flow cytometric calcium measurements. The dark grey graphs represent the untreated controls, the white graphs the glutamate-treated samples, and the light grey areas the overlay of both graphs. **b** Here, the right shift in Rhod-2 fluorescence in response to 11 mM glutamate was quantified. The bar graph shows one representative experiment with three replicates per sample (mean + SD; 10,000 cells per replicate). ***p < 0.001 compared to glutamate-treated ctrl (ANOVA, Scheffé’s-test)

Importantly, the detrimental mitochondrial Ca^2+^ overload constitutes a central event during programmed cell death and is tightly coupled to excessive mitochondrial ROS production^33^. Here, we assessed mitochondrial calcium concentrations using the fluorescent dye rhodamine-2. The fluorescence intensity of rhodamine-2 increases upon binding calcium and the quantification of gated cells displays that the glutamate-induced rise in mitochondrial calcium was fully blocked in *Cacna1c* siRNA-transfected cells compared to the controls (Fig. 4a,b). This effect of *Cacna1c* siRNA could be attributed to lower levels of Ca_V_1.2 in the cell membrane, which may contribute to a reduced influx of exogenous Ca^2+^, thereby positively influencing the deregulated calcium homeostasis in this model of oxidative stress^34^. This conclusion was further supported by the effects of the dihydropyridine (DHP) calcium channel blocker nimodipine, which protected HT22 cells against glutamate-induced mitochondrial calcium overload at a concentration of 100 µM similar to the effects achieved by *Cacna1c* knockdown (Supplementary Fig. S6a). To further investigate the mechanisms linking *Cacna1c* downregulation to mitochondrial calcium load we next assessed the cellular localization of Ca_V_1.2 in HT22 cells (Supplementary Fig. S7a). Consistent with existing evidence describing a plasma membrane localization of voltage-gated calcium channels, we found that Ca_V_1.2 was mostly present in the total lysate and to a lesser extent in the cytosolic fraction. As expected, Ca_V_1.2 was not appreciably expressed in the mitochondrial fraction rather excluding a direct effect of *Cacna1c* gene silencing on mitochondrial calcium concentration. Therefore, we then evaluated if *Cacna1c* knockdown indirectly affects the expression of the mitochondrial calcium uniporter (MCU; Supplementary Fig. S7b,c). The MCU is an inner mitochondrial membrane transport protein and essential for the regulation of calcium uptake. Under control conditions, oxidative glutamate toxicity caused an increase in MCU levels after 16 h. This glutamate-induced rise in MCU expression was significantly abolished in both *Cacna1c* siRNA-transfected and nimodipine-treated cells, thereby correlating well with our findings from the Rhod-2 calcium measurements. Furthermore, these results are in line with a previous study showing that knockdown of MCU in mouse neurons reduces NMDA-induced increases in mitochondrial calcium resulting in resistance to excitotoxicity^35^. Overall, these results demonstrate that both *Cacna1c* gene silencing and pharmacological inhibition of L-type calcium channels (LTCCs) provided a consistent protection of different mitochondrial parameters in paradigms of oxidative glutamate neurotoxicity.

### Knockdown of *Cacna1c* mediated protection against glutamate-induced cell death

**Fig. 5:**
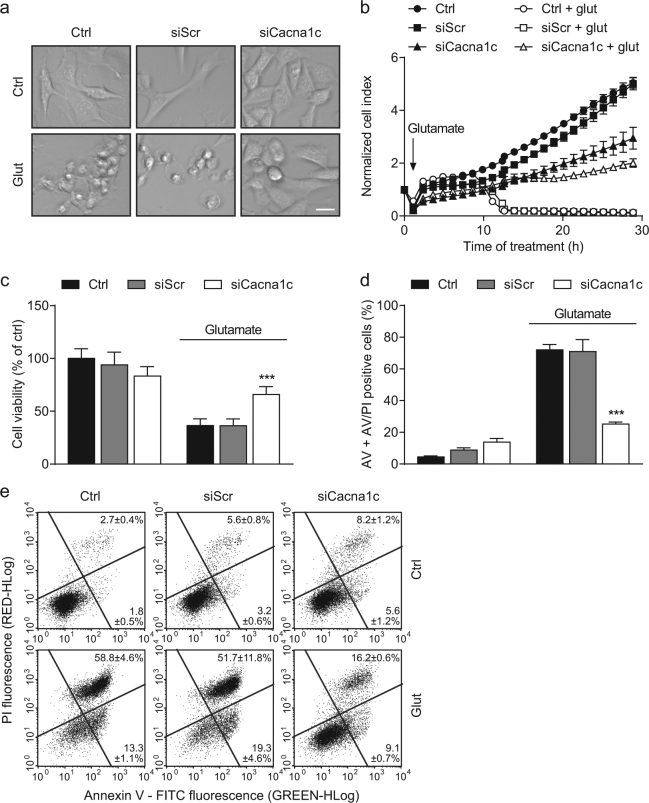
*Cacna1c* silencing protected HT22 cells from glutamate-induced cell death. **a** Representative phase-contrast images of glutamate-treated HT22 cells illustrate changes in cell morphology. Scale bar 25 µm. **b** A representative real time cell impedance measurement over 30 h after the treatment with 8 mM glutamate is shown. Eight replicate wells per condition are displayed as mean ± SD. **c** Before cell viability was determined by MTT assay, HT22 cells were challenged with 8 mM glutamate for 16 h. Values from 8 wells per condition are presented as mean + SD. The absorbance under control conditions is set as 100%. **d** The bar graph shows the percentage of annexin V and AV/PI positive cells. One representative experiment with three replicates per sample is depicted (mean + SD; 10,000 cells per replicate). **e** In addition, the dot plots exemplify the subdivision into AV (lower right quarter) and AV/PI positive cells (upper right quarter). AV annexin V, PI propidium iodide. ***p < 0.001 compared to glutamate-treated ctrl (ANOVA, Scheffé’s-test)

The positive effects of *Cacna1c* depletion in the applied model of oxidative stress were also reflected at the level of cell morphology and viability. Representative images of glutamate-treated controls showed characteristic features of dying cells, which were shrinking, rounding up, and detaching from the culture dish. In contrast, healthy and viable cells appeared elongated and well attached to the well bottom, as detected in the untreated control and glutamate-exposed siCacna1c conditions (Fig. 5a).

Finally, we investigated cell proliferation and viability in real time via continuous electrical impedance readout and found that, compared to controls, *Cacna1c* siRNA-transfected cells showed a significantly lower proliferation rate under basal conditions (Fig. 5b). As cell proliferation is tightly coupled to energy metabolism, this effect may be due to the before mentioned siCacna1c-mediated inhibition of the mitochondrial electron transport chain^36^. However, *Cacna1c* silencing mediated persistent protection of HT22 cells from glutamate toxicity. On the contrary, in the glutamate-treated controls a fast decrease in the normalized cell index was observed after 12 to 15 h of glutamate administration, indicating cell death. In line with this result from real time impedance measurements, the protective effect of the *Cacna1c* siRNA was also detected in the MTT reduction assay after 16 h of glutamate exposure (Fig. 5c). Both findings were independently confirmed by an additional siRNA sequence (siCacna1c 3; Supplementary Fig. S4b,c). Accordingly, flow cytometric measurements using annexin V and propidium iodide (PI) provided concurrent results. The two dyes stain early apoptotic and late apoptotic/necrotic cells respectively (Fig. 5d,e). The quantification presented in Fig. 5d indicates a significantly enhanced resistance against glutamate-induced cell death in *Cacna1c* siRNA-transfected cells. Supporting these results, nimodipine (100 µM) also protected the cells from glutamate toxicity as shown by the MTT assay and annexin V/PI staining (Supplementary Fig. S6b,c).

## Discussion

In the present study, we found that both siRNA-mediated downregulation and pharmacological inhibition of *Cacna1c* in neuronal HT22 cells mediated substantial protective effects on lipid peroxidation, mitochondrial integrity and function, and cell viability in a model of glutamate-induced cell death. These results suggest that, in paradigms of oxidative stress, *Cacna1c* is significantly involved in mechanisms of neuronal damage.

Our findings may also be of potential relevance in human diseases, since hippocampal and cortical atrophy, which are closely associated with abnormalities in neuronal plasticity and reduced resilience to cellular stress, have been frequently reported in patients with psychiatric disorders and early-life adverse experiences^37,38^. Furthermore, recent studies revealed that healthy subjects carrying the *CACNA1C* risk variant rs1006737 also show decreased prefrontal cortex activation and hippocampal dysfunction^39,40^. Even though it is not consistently observed^41,42^, the majority of evidence suggests that the non-coding risk SNP rs1006737 is associated with enhanced *CACNA1C* expression and a gain of Ca_V_1.2 function^10,12,43^, suggesting deregulated Ca^2+^ signaling in the affected population and a significantly increased probability of psychiatric diseases^13^. These findings are further confirmed by several animal studies demonstrating that, besides an increase in anxiety-associated behavior^44^, *Cacna1c* heterozygous and forebrain-specific knockout mice exhibit an antidepressant-like phenotype, resilience to chronic stress, and a protective phenotype against mania-related behavior^45–48^. In addition to the data based on genetic modifications, the pharmacological inhibition of L-type calcium channels has repeatedly been linked to anti-stress and antidepressant-like effects, and reduced despair-like behavior in rodents^49–51^. Moreover, dihydropyridine compounds with good central nervous system (CNS) penetration, such as nimodipine showed promising mood stabilizing effects in humans with affective disorders, suggesting that LTCC antagonists represent an additional option in the pharmacotherapy of psychiatric diseases, and especially patients carrying the *CACNA1C* risk SNP could benefit from this alternative treatment strategy^13,43,52^. Elevated cytosolic calcium levels, which may be associated with the *CACNA1C* risk SNP rs 1006737, represent a major upstream pathway that impairs mitochondrial function in BD^53^. In this regard, it has been reviewed recently that systemic administration of the DHP isradipine suppresses cytosolic calcium transients, increases mitochondrial mass and lowers mitophagy in dopaminergic neurons^54^. These findings in vivo are in line with our own results in vitro, revealing an enhanced resistance against oxidative stress and beneficial effects on mitochondrial function in conditions of reduced *Cacna1c* expression and LTCC blockade with nimodipine.

As reported previously, impaired cellular adaptation to environmental stress constitutes another major trigger in the pathophysiology of psychiatric disorders^2^. In particular, it has been demonstrated in animal models that psychosocial stress results in depression-like behavior through the activation of oxidative and apoptotic mechanisms, including increased ROS levels, lipid peroxidation, reduced Bcl-2 expression, and increased caspase-3^22^. Additionally, in human post-mortem studies a downregulation of several antioxidant genes and an increase in protein oxidation have been observed in the hippocampus and prefrontal cortex of patients with BD^55,56^. Hence, the applied model of glutamate-induced oxidative stress in hippocampal HT22 cells constitutes a suitable system reflecting a common cellular response to environmental stress in the development and course of psychiatric illnesses.

It has been repeatedly proposed that interactions between genetic and environmental risk factors significantly contribute to the complex and heterogeneous clinical manifestations of affective disorders^2^. Our study provides valuable insights into a gene-stress-interaction by showing that reduced *Cacna1c* expression, which represents a beneficial genetic factor, mediated neuroprotective effects against oxidative stress, particularly at the level of mitochondria. Mitochondrial function is essential for cellular resilience and adaptation to oxidative stress. Here, siRNA-mediated knockdown of *Cacna1c* preserved mitochondrial morphology, ATP values, respiration, membrane potential, and calcium homeostasis, and, furthermore, diminished excessive ROS formation in the employed model of oxidative glutamate toxicity. These findings are highly relevant as increasing evidence suggests a crucial role for mitochondrial dysfunction and related key determinants of cellular stress, such as intracellular Ca^2+^ deregulation and ROS formation in the development of mood disorders^24^.

Furthermore, our findings indicate a possible link between *Cacna1c* expression and mitochondrial function in oxytosis. In this paradigm of oxidative cell death, we found that both *Cacna1c* gene silencing and pharmacological inhibition of LTCC using nimodipine significantly diminished the glutamate-induced rise in mitochondrial calcium levels. We conclude that this effect was attributed to a reduced cellular calcium influx, thereby leading to a mild inhibition of complex IV and also affecting the downstream mitochondrial Ca^2+^ influx. We showed previously, however, that Ca^2+^ influx through the plasma membrane only partially contributed to mitochondrial damage and cell death in the applied model of glutamate-induced oxidative stress^57^. Based on the pronounced protective effects of *Cacna1c* knockdown and LTCC blockade, it has to be taken into consideration that, beyond its influence on calcium homeostasis at the plasma membrane, *Cacna1c* is possibly involved in further regulatory pathways at the level of mitochondria. For example, the calcium-dependent cytosolic phosphatase calcineurin dephosphorylates Drp1 thereby promoting its translocation to mitochondria and triggering detrimental mitochondrial fission; a process that is likely inhibited by *Cacna1c* gene silencing^58^. Moreover, both *Cacna1c* knockdown and LTCC blockade probably lead to altered Ca_V_1.2-dependent gene transcription regulation and may suppress toxic mitochondrial Ca^2+^ uptake via transcriptional repression of enhanced MCU expression following glutamate treatment in HT22 cells^35^. Conversely, it has been described in arterial myocytes that mitochondria stimulate NF-κB-dependent Ca_V_1.2 expression via elevated mitochondrial calcium concentration and subsequently induced mitochondrial ROS generation^59^. In this regard, it is suggested that silencing of *Cacna1c* possibly counteracts the respective feed-forward signaling cascade. In mast cells, however, *Cacna1c* knockdown augmented apoptosis after IgE stimulation by disrupting mitochondrial integrity, emphasizing that the maintenance of an appropriate mitochondrial calcium concentration is also essential for cell survival^60^.

We also observed that *Cacna1c* downregulation led to a reduced formation of lipid peroxides in HT22 cells upon glutamate exposure, indicating a partial action upstream of mitochondria. But since mitochondrial damage acts as an amplification factor of lipid peroxidation, mitochondria might still represent the main point of action of *Cacna1c* silencing in this model of oxidative glutamate toxicity^26^.

Overall, our findings suggest that the GWAS-confirmed psychiatric risk gene *CACNA1C* plays a major role in oxidative stress pathways with particular impact on mitochondrial integrity and function. However, the precise molecular mechanisms underlying the effects of *CACNA1C* regulation on mitochondrial performance in neurons and the emerging link to neuronal plasticity, maintenance and function in the context of affective disorders remain to be elucidated.

## Materials and methods

### Cell culture

HT22 cells were immortalized from primary hippocampal neurons using a temperature-sensitive SV-40 T antigen and selected from HT4 cells based on sensitivity to glutamate^61,62^. Since HT22 cells do not express functional ionotropic glutamate receptors, glutamate-induced cell death is mediated via an oxidative stress-dependent pathway called oxytosis. Oxidative glutamate toxicity in HT22 cells involves the inhibition of the glutamate/cystine antiporter, a subsequent depletion of glutathione, an increase in ROS production, a consecutive impairment of mitochondria, and an influx of Ca^2+^, which ultimately leads to neuronal cell death^25–27,31^.

HT22 cells were cultured at 37 °C and 5% CO_2_ in Dulbecco’s modified Eagle’s medium (DMEM; Sigma-Aldrich, Munich, Germany) supplemented with 10% fetal bovine serum, 20 mM HEPES, 100 units/ml penicillin, 100 µg/ml streptomycin, and 2 mM glutamine. The cells were transfected with an optimized concentration of 40 nM siRNA using Lipofectamine RNAiMAX (Thermo Fisher Scientific, Darmstadt, Germany) in antibiotic-free growth medium. The siRNA sequences were as follows: scrambled *Cacna1c* siRNA (siScr, 5′-AUUGGCGCACUAUUAGACU-3′), *Cacna1c* siRNA (siCacna1c, 5′-GGAAAGCUCUAUACCUGUU-3′). The plated cells were treated with millimolar concentrations of glutamate for 15 to 18 h unless otherwise stated and assayed 30 to 48 h after transfection. The L-type calcium channel (LTCC) blocker nimodipine (100 mM stock solution in DMSO; Cayman Chemical, Ann Arbor, Michigan, USA) was used in a final concentration of 100 µM and applied together with 6 to 8 mM glutamate for 16 h.

### RT-PCR

HT22 cells were seeded at a density of 180,000 cells/well in 6-well plates. Total RNA was isolated with the InviTrap Spin Universal RNA Mini Kit (Stratec Biomedical, Birkenfeld, Germany) 24 h after siRNA transfection. Reverse transcription PCR (RT-PCR) was performed using SuperScript III One-Step RT-PCR System (Thermo Fisher Scientific, Darmstadt, Germany) and the following primers: *Cacna1c* (502 bp) forward 5′-CAACGCAACCATCTCTACCG-3′ and reverse 5′-CAGTGCCTTCACGTC-GAATC-3′; *Gapdh* (399 bp) forward 5′-CGTCTTCACCACCATGGAGAAGGC-3′ and reverse 5′-AAGGCCATGCCAGTGAGCTTCCC-3′. The amplified products were visualized under UV light on a 1.5% agarose gel.

### Protein analysis

Protein extraction and Western blot were performed according to established protocols^31^. Briefly, cells were lysed 48 h after transfection in 0.25 M D-mannitol, 0.05 M Tris base, 1 mM EDTA, 1 mM EGTA, 1 mM DTT, and 1% Triton X-100 supplemented with protease and phosphatase inhibitor cocktail tablets (Roche Diagnostics, Mannheim, Germany). The total protein amount was determined using the Pierce BCA Protein Assay Kit (Thermo Fisher Scientific, Darmstadt, Germany) and 60 µg protein were loaded on a 7.5% polyacrylamide gel. After electrophoresis, the proteins were transferred from the gel onto a PVDF membrane (Roche Diagnostics, Mannheim, Germany). Then, the membranes were incubated with anti-Ca_V_1.2 antibody (1:200; Alomone Labs, Jerusalem, Israel). Vinculin was detected as a loading control using an anti-Vinculin antibody (1:20,000; Sigma-Aldrich, Munich, Germany).

### Determination of total glutathione

The amount of total glutathione in HT22 cells was quantified using the luminescence-based GSH/GSSG-Glo Assay (Promega, Mannheim, Germany) in accordance with the manufacturer’s instructions. HT22 cells were plated in a density of 6,000 cells/well in white-with-clear-bottom 96-well plates. Twenty-four hours after seeding, the cells were incubated for 2, 4, and 6 h with 10 mM glutamate. Absolute glutathione concentrations were calculated from a GSH standard curve.

### Mitochondrial morphology

The evaluation of mitochondrial morphology was carried out as reported and validated previously with minor modifications^25^. In brief, 14,000 cells/well were seeded in 8-well ibiTreat plates (Ibidi, Martinsried, Germany). To visualize mitochondria, cells were incubated with 0.2 µM MitoTracker Deep Red (Thermo Fisher Scientific, Darmstadt, Germany) for 30 min before adding glutamate. After the fixation with 4% paraformaldehyde, images were acquired using a fluorescence microscope (DMI6000 B; Leica Microsystems, Wetzlar, Germany) at a 63× magnification. To generate reliable results according to previous validation, 500 cells per condition were counted and classified by their mitochondrial morphology into three different categories. Category 1 is defined by elongated and equally distributed mitochondria. Category 2 is characterized by partially fragmented mitochondria that are still distributed throughout the cytosol. Finally, Category 3 is represented by strongly fragmented mitochondria that are located around the nucleus. Three independent experiments were performed and the investigator was always blinded to the treatment history.

### ATP measurement

The determination of ATP levels was performed using the ViaLight Plus Kit (Lonza, Verviers, Belgium) according to the manufacturer’s protocol. Briefly, cells were lysed, transferred to a white-walled 96-well plate and the luciferase containing ATP monitoring reagent was added. The resulting luminescence was detected with a FLUOstar OPTIMA reader (BMG Labtech, Ortenberg, Germany).

### Measurement of OCR

The OCR as an indicator of mitochondrial respiration was measured with a Seahorse XF^e^96 Analyzer (Agilent Technologies, Waldbronn, Germany). The system allows for the consecutive injection of different modulators of the electron transport chain. The compounds and final concentrations used are as follows: oligomycin 3 µM, FCCP 0.5 µM, rotenone 0.1 µM, and antimycin A 1 µM. Three basal and three measurements after each injection were recorded. HT22 cells were assayed in DMEM containing 25 mM D-glucose, 2 mM glutamine, and 1 mM sodium pyruvate with an adjusted pH of 7.35.

### Flow cytometry

Several parameters associated with glutamate-induced oxidative stress, such as lipid peroxidation, mitochondrial ROS formation, mitochondrial membrane potential, mitochondrial Ca^2+^ levels, and cell death, were analyzed with a Guava easyCyte 6–2 L flow cytometer (Merck Millipore, Darmstadt, Germany) using different fluorescent dyes. HT22 cells were plated at 30,000–55,000 cells/well in a 24-well format. Measurements were performed in triplicates, each with a total count of 10,000 cells, and are representative of a least three independent experiments.

#### Lipid peroxidation

BODIPY 581/591 C11 (4,4-difluoro-5-(4-phenyl-1,3-butadienyl)-4-bora-3a,4a-diaza-s-indacene-3-undecanoic acid; Thermo Fisher Scientific, Darmstadt, Germany) was utilized as a sensor for the oxidation of lipids and membranes. Here, HT22 cells were treated with 9 mM glutamate 24 h after siRNA transfection. Following 8 h of glutamate treatment, the cells were stained with 2 µM BODIPY dye for 1 h at 37 °C.

#### Mitochondrial superoxide formation

Superoxides are the predominant reactive oxygen species in mitochondria and were detected by incubation of the cells with 1.25 µM MitoSOX Red indicator (Thermo Fisher Scientific, Darmstadt, Germany) for 30 min at 37 °C^27^.

#### Mitochondrial membrane potential

Mitochondrial membrane depolarization was determined using the MitoPT TMRE Kit (ImmunoChemistry Technologies, Hamburg, Germany) following the recommendations of the manufacturer. HT22 cells were collected and stained with 0.2 µM TMRE (tetramethylrhodamine ethyl ester) for 30 min at 37 °C. The depolarizing reagent CCCP (carbonyl cyanide 3-chlorophenylhydrazone) was used in a final concentration of 50 µM and served as additional positive control.

#### Mitochondrial calcium concentration

To selectively study mitochondrial calcium levels Rhod-2 AM (rhodamine-2 acetoxymethyl ester; Thermo Fisher Scientific, Darmstadt, Germany) was reduced to Dihydrorhod-2 AM. Then, HT22 cells were harvested and incubated with 2 µM of the reduced dye in DMEM without serum for 30 min.

#### Cell death

Cell death was evaluated using the Annexin V-FITC Detection Kit (Promokine, Heidelberg, Germany) in accordance with the manufacturer’s instructions. The two containing dyes Annexin V and PI stain early apoptotic and dead cells respectively. Shortly, HT22 cells were trypsinized and the cell suspensions incubated with 5 µl/ml of each dye for 5 min at room temperature.

### Cell viability

Cell viability was assessed by a colorimetric assay where MTT (3-(4,5-dimethyl-2-thiazolyl)-2,5-diphenyl-2H-tetrazolium bromide, 0.5 mg/ml; Sigma-Aldrich, Munich, Germany) is NADH-dependently reduced to a purple colored formazan product, which is then quantified by absorbance measurement at 570 nm with a reference filter at 630 nm.

As described previously, the xCELLigence Real-Time Cell Analysis (RTCA; Roche Diagnostics, Mannheim, Germany) system allows cell viability to be measured continuously in real time via electrical impedance readout^63^. Briefly, attached and proliferating cells cause a high and further increasing impedance signal, whereas the detachment of dying cells leads to a decrease in electrical impedance. Changes in the impedance are depicted as normalized cell index. For both assays HT22 cells were seeded in 96-well plates at 6,000-8,000 cells per well.

### Statistical analysis

All data are presented as mean values with standard deviation (SD). Multiple comparisons were performed by one-way analysis of variance (one-way ANOVA) followed by Scheffé’s post hoc test. All experiments were performed in 3 to 8 replicates per treatment condition, as indicated, and were independently repeated at least twice for confirmation of the results. The parameters presented were calculated using the WinSTAT statistics Add-In for Microsoft Excel (R. Fitch Software, Bad Krozingen, Germany). A statistical significant difference was assumed at *p* < 0.05 (*), *p* < 0.01 (**), and *p* < 0.001 (***).

## Electronic supplementary material

Supplementary information
