# Supplementary material for: Downregulation of the psychiatric susceptibility gene Cacna1c promotes mitochondrial resilience to oxidative stress in neuronal cells
Source: Cell Death Discov. 2018 May 10;4:54. doi: 10.1038/s41420-018-0061-6 (PMC5945680; doi:10.1038/s41420-018-0061-6)
Supplement: Supplementary file 1 — Supplementary information [file 41420_2018_61_MOESM1_ESM.pdf]

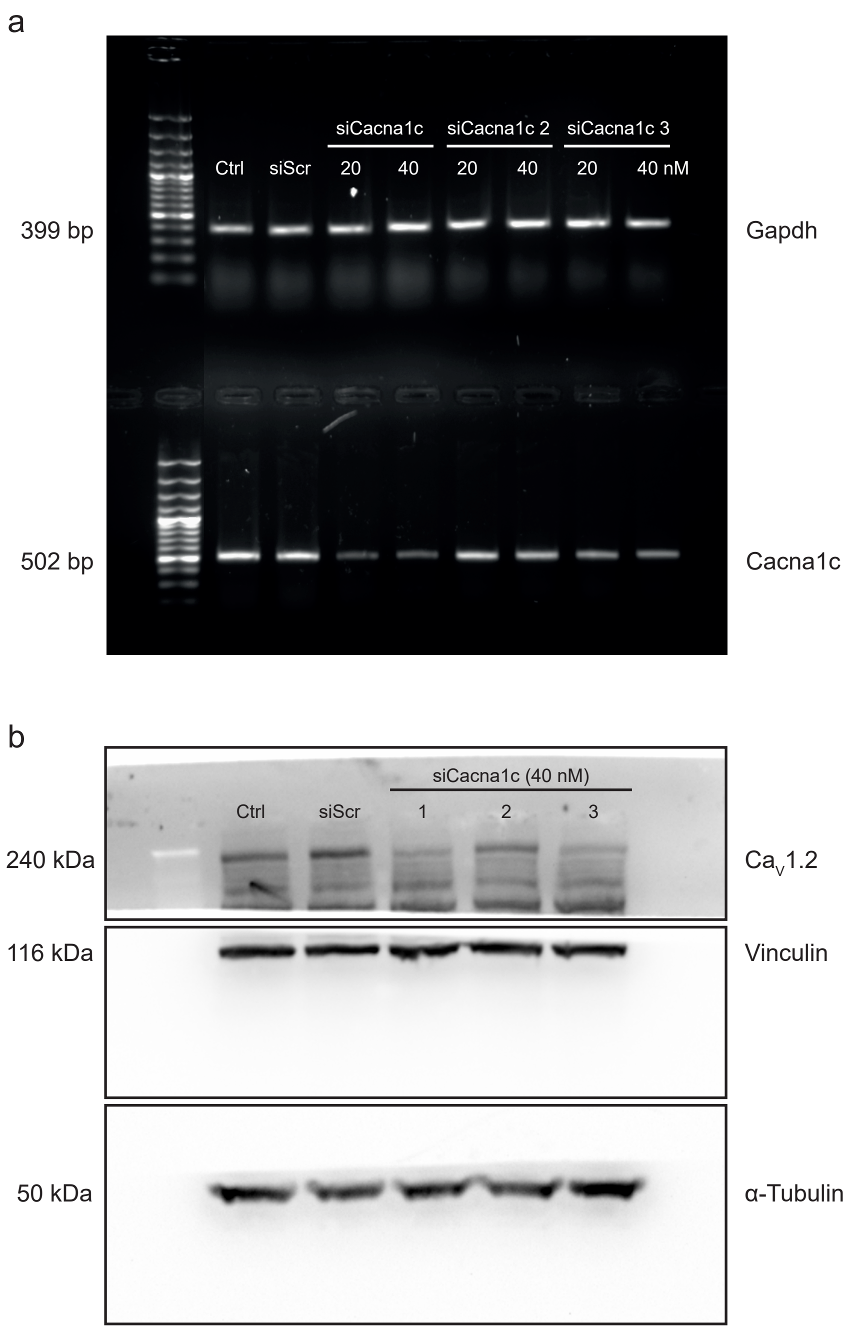

Figure S1 Effects of Cacna1c siRNA transfection on Cacna1c mRNA and protein levels. (a) *Cacna1c* mRNA levels from three different *Cacna1c* siRNAs were analyzed 24 h after siRNA transfection using *Gapdh* as internal control. (b) Protein samples were collected 48 h after transfection with 40 nM siRNA and the $Ca_V1.2$ expression levels were then identified by Western blot. Vinculin and α-Tubulin are used as loading control.

The sequences of the supporting two siRNAs were as follows:

siCacna1c 2 (5‘-CUAGUAUCUUUACAUUAGA-3‘),

siCacna1c 3 (5‘-GUGCCACCGUAUUGUCAAU-3‘).

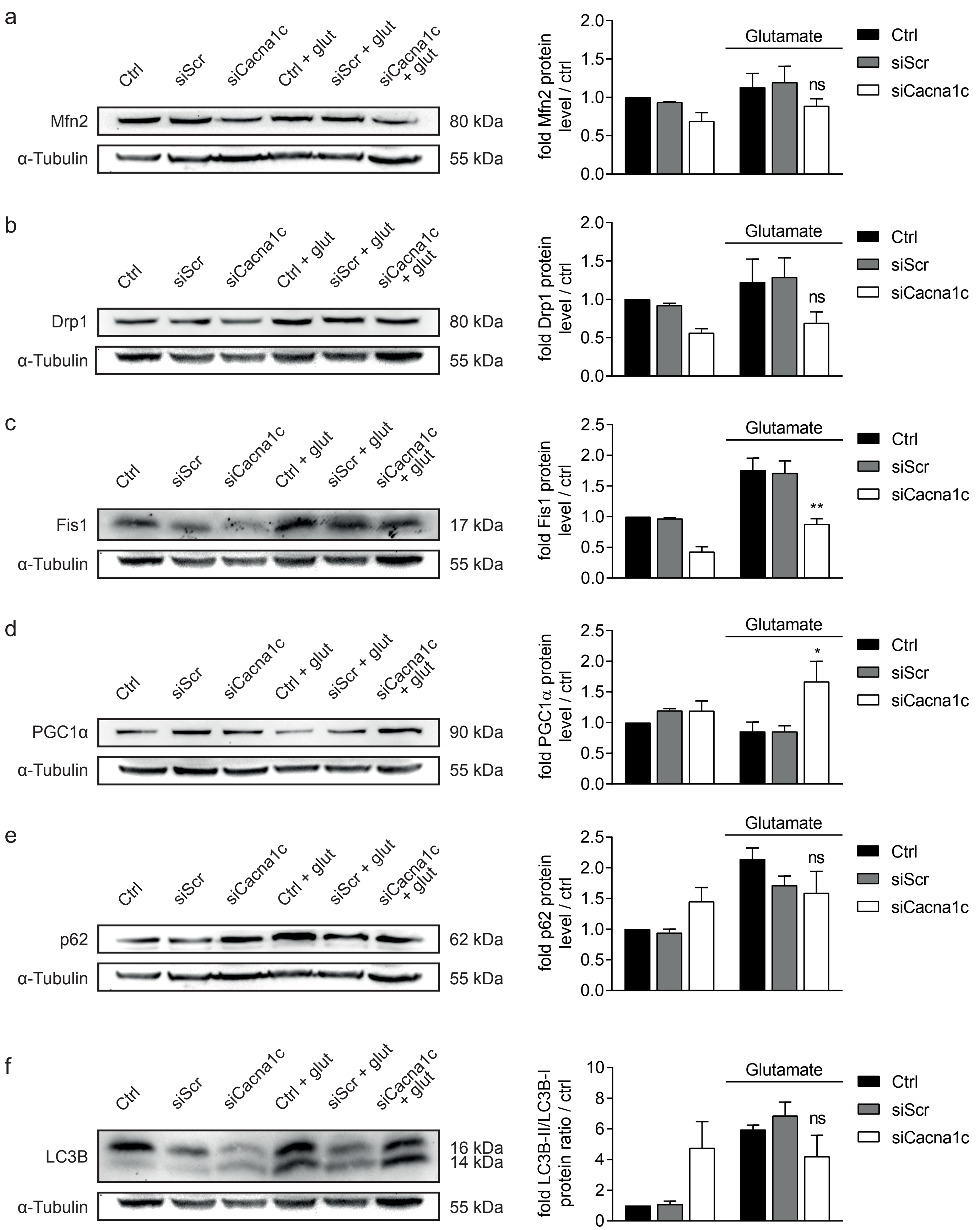

Figure S2 *Cacna1c* gene silencing led to diminished mitochondrial fission and increased mitochondrial biogenesis following glutamate challenge.

After 16 h of glutamate treatment, the expression levels of the mitochondrial fusion protein Mfn2 (a), the fission proteins Drp1 and Fis1 (b,c), the mitochondrial biogenesis marker PGC1α (d) and the mitophagy indicators p62 and LC3B-II (e,f) were identified by Western blot. One representative immunoblot per protein is shown (left panel). The bar graphs (right panel) were obtained by densitometric quantification of the Western blot data and normalization to α-Tubulin. Data are presented as mean + SEM (n = 3-4). Ctrl, control; siScr, scrambled siRNA; siCacna1c, *Cacna1c* siRNA; Glut, glutamate. **p<0.01; *p<0.05; ns (not significant) compared to glutamate-treated ctrl (ANOVA, Scheffé's-test).

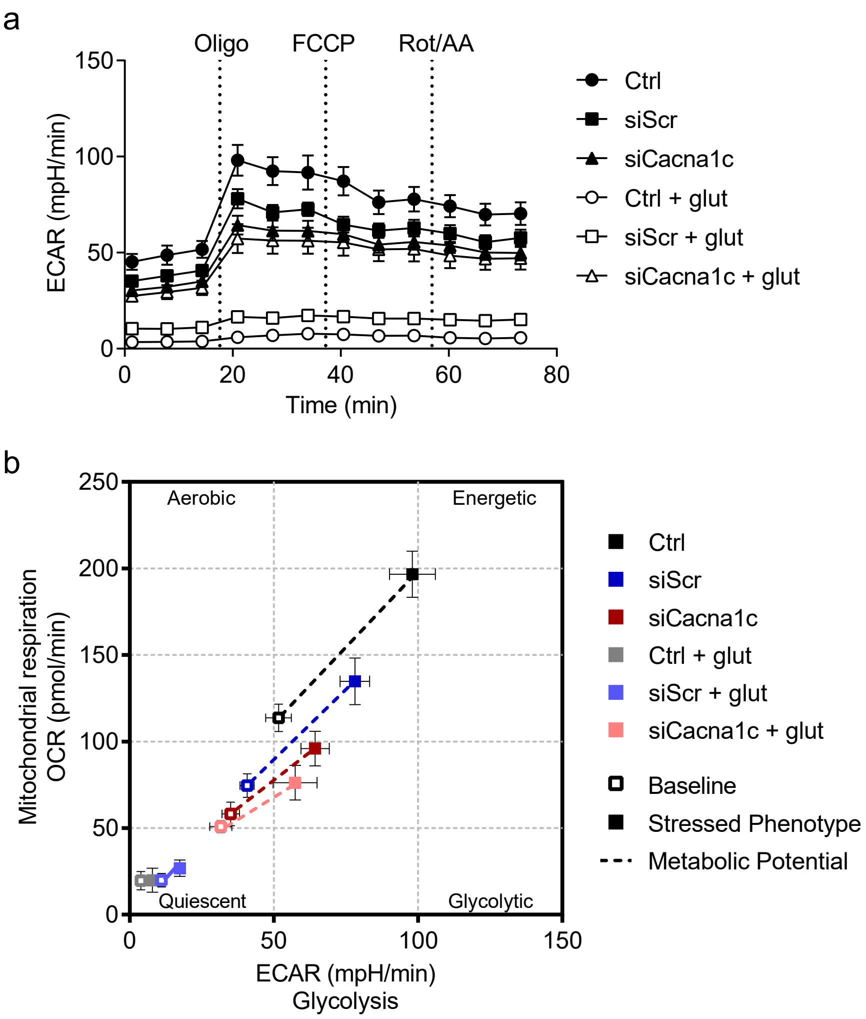

Figure S3

(a) After 16 h of glutamate challenge, the extracellular acidification rate (ECAR) was determined with a Seahorse XFe96 Analyzer. Data of 3-7 replicate wells per condition are given as mean ± SD. Oligo, oligomycin; FCCP, carbonyl cyanide 4-(trifluoromethoxy) phenylhydrazone; Rot, rotenone; AA, antimycin A. (b) In the cell energy phenotype graph the OCR is plotted against the ECAR. The baseline phenotype (open square) represents the OCR and ECAR of HT22 cells at starting assay conditions. The stressed phenotype (filled square) displays the OCR and ECAR under an induced energy demand, i.e. in the presence of the two stressors oligomycin and FCCP. The resulting metabolic potential (dashed line) reflects the ability to meet an induced energy demand.

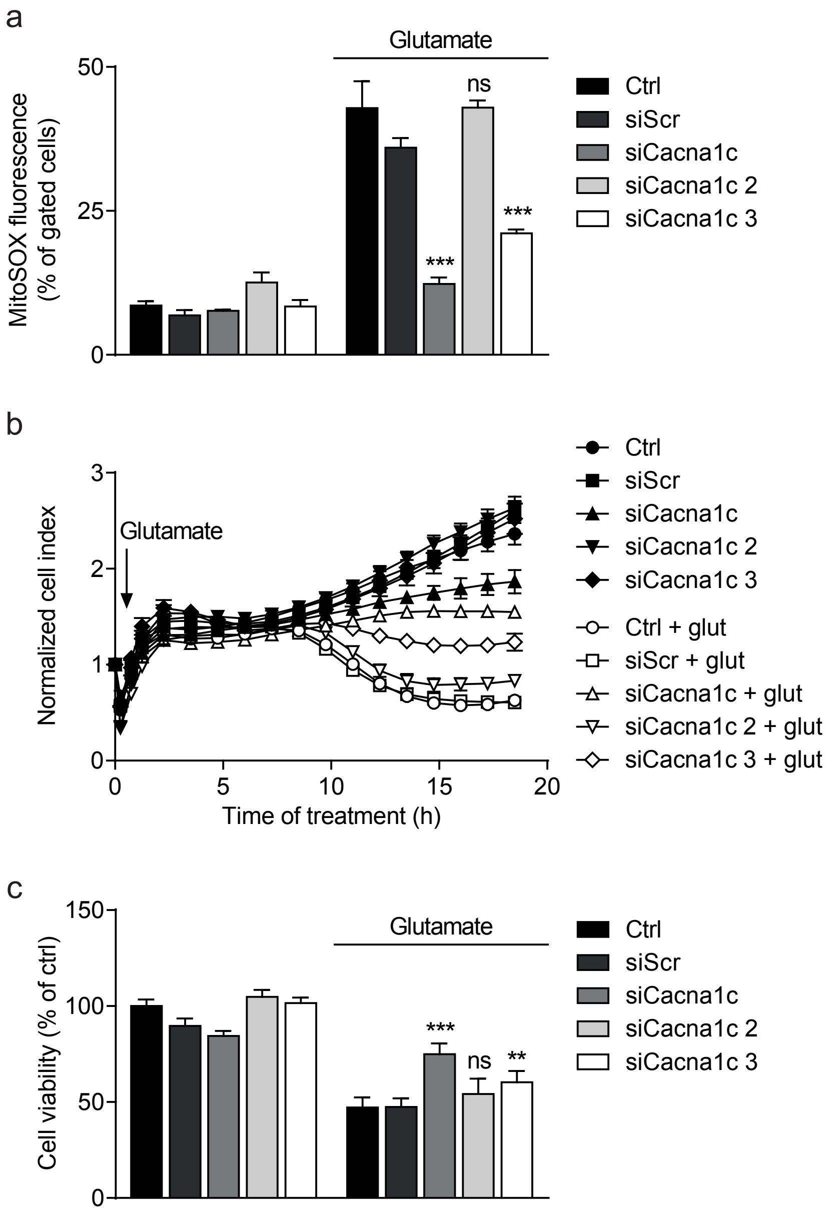

Figure S4 Only a pronounced knockdown of Cacna1c mediated protective effects against glutamate toxicity in HT22 cells.
(a) Mitochondrial superoxide formation was assessed by flow cytometry using 1.25 µM MitoSOX. The bar graph shows one representative experiment with three replicates per sample (mean + SD; 10 000 cells per replicate). (b) xCELLigence measurements were conducted after the treatment with 10 mM glutamate. Three to six replicate wells per condition are displayed as mean ± SD. (c) Cell viability was evaluated in glutamate-challenged HT22 cells by measuring MTT formazan absorbance (9 mM, 17 h). Eight replicate wells per condition are displayed as mean + SD. The absorbance under control conditions is set as 100%. ***$p<0.001$; **$p<0.01$; ns (not significant) compared to glutamate-treated ctrl (ANOVA, Scheffé's-test).

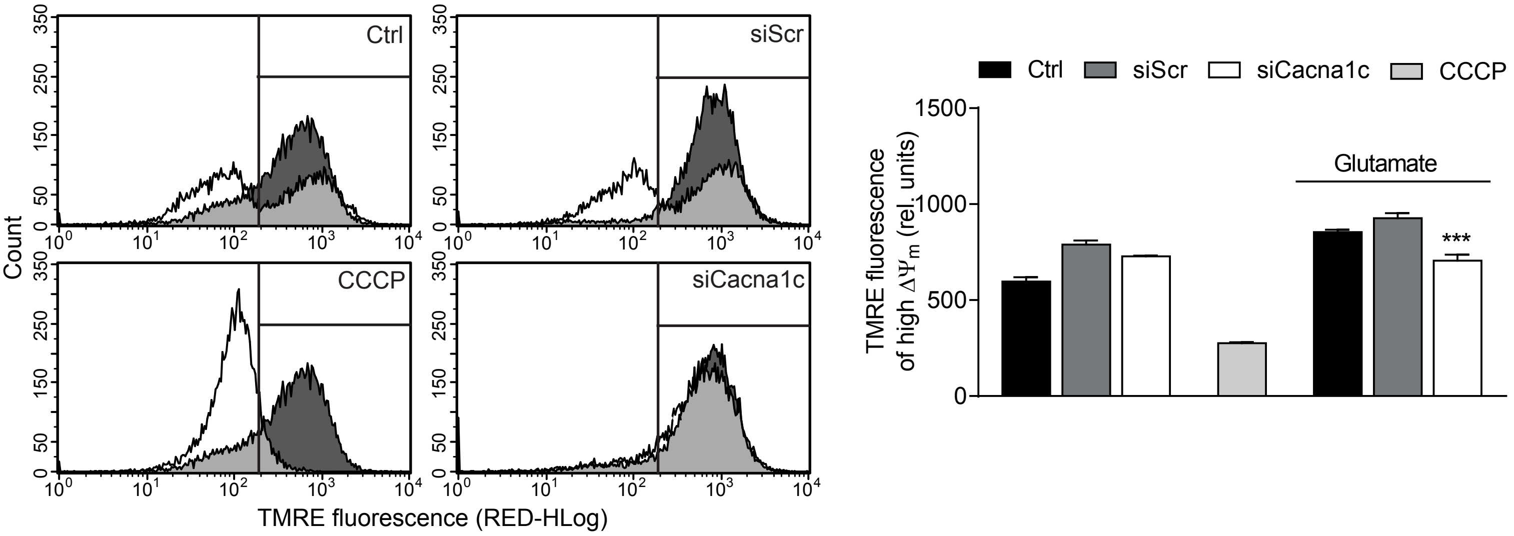

Figure S5
The mitochondrial membrane potential was analyzed by flow cytometry using the voltage-sensitive fluorescent dye TMRE. In the histograms, the dark grey graphs represent the untreated conditions, the white graphs the glutamate-treated samples, and the light grey areas the overlay of both graphs. CCCP (carbonyl cyanide 3-chlorophenylhydrazone; 50 µM) is a mitochondrial membrane depolarizer and serves as positive control. Mitochondrial membrane depolarization leads to a left shift in TMRE fluorescence, whereas $\Delta\Psi_m$ hyperpolarization results in a right shift. The bar graph shows one representative experiment with three replicates per sample (mean + SD). ***$p<0.001$ compared to glutamate-treated ctrl (ANOVA, Scheffé‘s-test).

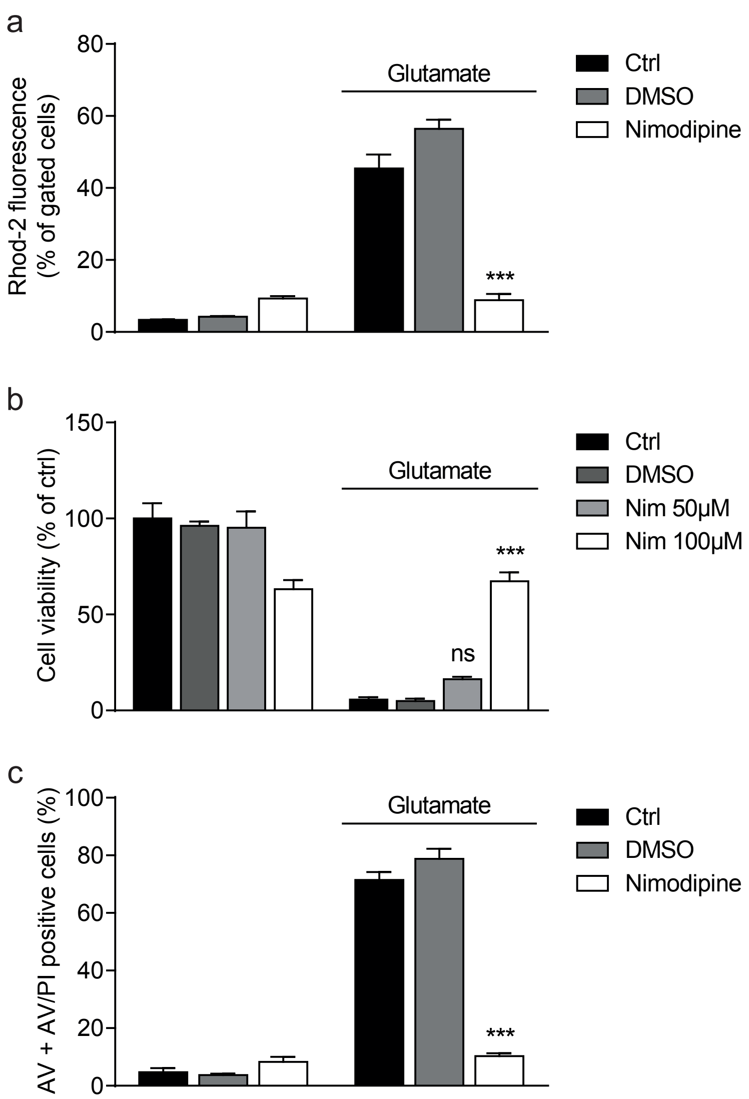

Figure S6 The L-type calcium channel blocker nimodipine ameliorated mitochondrial function and cell survival under glutamate treatment.

(a) Mitochondrial calcium levels were investigated by flow cytometry using Rhod-2. HT22 cells were co-treated with 100 µM nimodipine and 6 mM glutamate for 16 h. The bar graph depicts one representative experiment with three replicates per sample (mean + SD; 10 000 cells per replicate). (b) Cell viability was assessed after 16 h of glutamate and nimodipine exposure by a colorimetric MTT assay. Six replicate wells per condition are displayed as mean + SD. The absorbance under control conditions is set as 100%. (c) The bar graph shows the percentage of annexin V and AV/PI positive cells after 16 h of glutamate (7 mM) and nimodipine (100 µM) co-treatment. One representative experiment with three replicates per sample is depicted (mean + SD; 10 000 cells per replicate). DMSO concentration in all experiments was 0.1%.

Nim, nimodipine; AV, annexin V; PI, propidium iodide. ***$p<0.001$; ns (not significant) compared to glutamate-treated ctrl (ANOVA, Scheffé's-test).

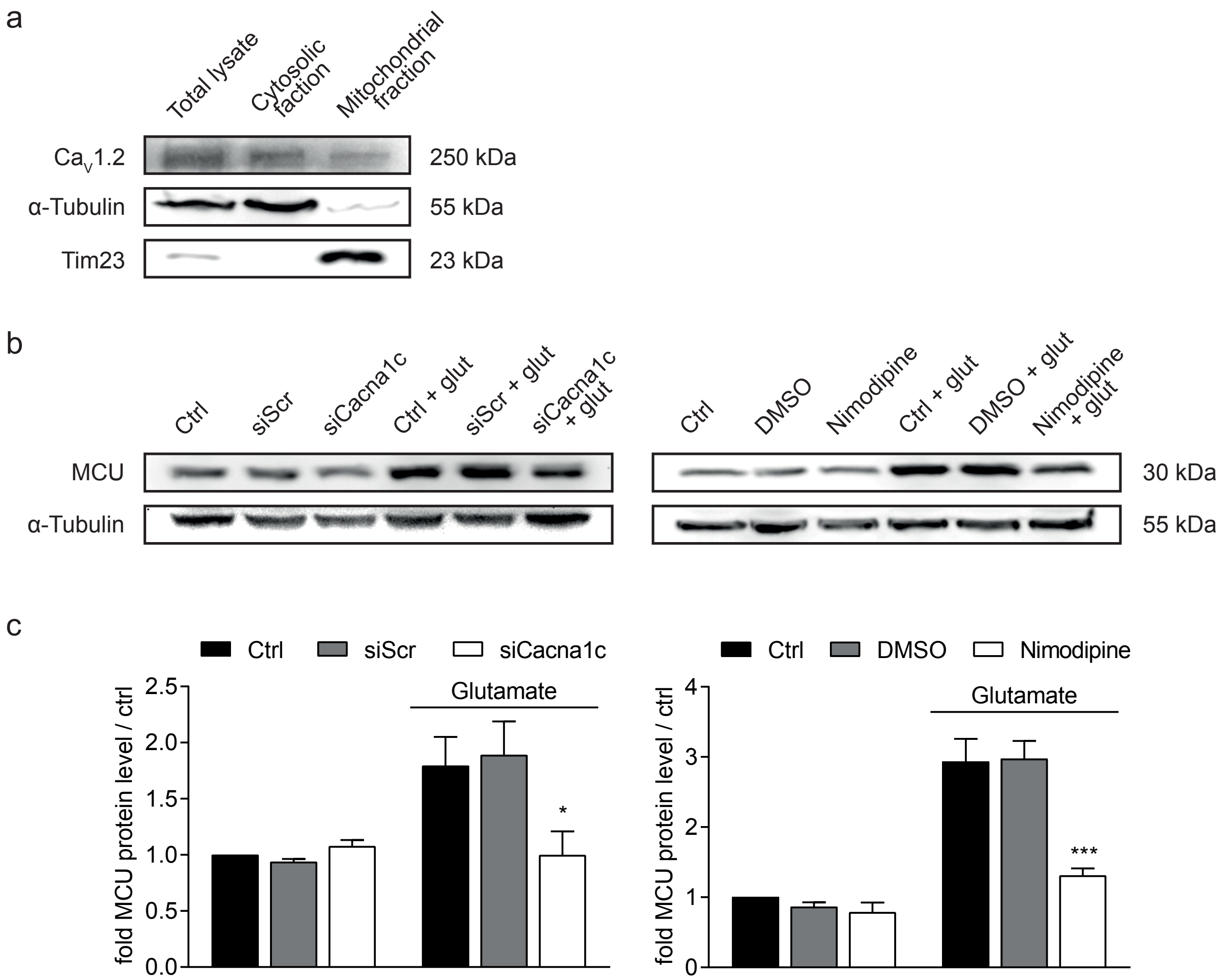

Figure S7

(a) Ca$_V$1.2 protein expression levels in the total lysate, the cytosolic and the mitochondrial fraction of HT22 cells were identified by Western blot. α-Tubulin was used as cytosolic marker protein. The inner mitochondrial membrane protein Tim23 served as indicator for mitochondrial enrichment. (b) The MCU expression levels in both *Cacna1c* siRNA-transfected and nimodipine-treated HT22 cells after 16 h of glutamate exposure were assessed by Western blot. One representative immunoblot per treatment condition is shown. (c) The relative MCU protein abundance was densitometrically quantified and α-Tubulin-normalized. Data are presented as mean + SEM (n = 4-5). Ctrl, control; siScr, scrambled siRNA; siCacna1c, *Cacna1c* siRNA; Glut, glutamate. ***p<0.001; *p<0.05 compared to glutamate-treated ctrl (ANOVA, Scheffé's-test).

Supplementary Materials and Methods
Protein analysis

Fifty microgram protein per sample were loaded on 12.5% polyacrylamide gels. After electrophoresis, the proteins were transferred onto a PVDF membrane (Roche Diagnostics, Mannheim, Germany) and incubated with the respective antibodies overnight at 4°C. Protein detection was realized using peroxidase labeled secondary antibodies (Vector Laboratories, Burlingame/California, USA) and luminol based HRP-Juice Plus (PJK GmbH, Kleinblittersdorf, Germany). The resulting chemiluminescence was imaged with a ChemiDoc XRS system (Bio-Rad Laboratories, Hercules/California, USA). Protein quantification was performed using the Bio-Rad Image Lab™ Software.

Antibody	Dilution	Mol. Wt. (kDa)	Host	Company
PGC1α	1:1,000	91	Rabbit	Rockland
Drp1	1:500	80	Mouse	BD Bioscience
Mfn2	1:1,000	80	Rabbit	Cell Signaling
p62	1:1,000	62	Rabbit	Cell Signaling
α-Tubulin	1:10,000	55	Mouse	Sigma
MCU	1:1,000	30	Rabbit	Cell Signaling
Fis1	1:500	17	Rabbit	Enzo Life Science
LC3B	1:500	14-16	Rabbit	Cell Signaling

Isolation of mitochondria from HT22 cells

Mitochondria were isolated using a pump controlled cell homogenizer (Isobiotec, Heidelberg, Germany) with a clearance of 10µm and a constant pump flow rate of 700 µl/min according to existing protocols (1). In brief, the trypsinized HT22 cell suspension was filled in a 1 ml gas tight glass syringe (Supelco, Munich, Germany) and homogenized with three strokes through the cell homogenizer resulting in the total lysate. Subsequently, the homogenate was centrifuged at 800 xg for ten minutes at 4°C. The supernatant was transferred to a fresh tube and centrifuged at 9,000 xg for ten minutes (Heraeus fresco 17, Thermo Fisher Scientific, Darmstadt, Germany). The emerging pellet represents the crude mitochondrial fraction and was slowly resuspended in 150 µl mitochondrial isolation buffer. The supernatant containing the cytosolic fraction was collected as well. The total protein concentration was determined using the Pierce BCA Protein Assay Kit (Thermo Scientific, Darmstadt, Germany) with BSA standards based on mitochondrial isolation buffer. Sixty microgram protein per fraction were separated on a two-phase polyacrylamide gel (7.5/15%).

Antibody	Dilution	Mol. Wt. (kDa)	Host	Company
$Ca_V1.2$	1:200	250	Rabbit	Alomone Labs
α-Tubulin	1:10,000	55	Mouse	Sigma
Tim23	1:2,000	23	Mouse	BD Bioscience

1. Schmitt S, Saathoff F, Meissner L, Schropp E-M, Lichtmannegger J, Schulz S et al. A semi-automated method for isolating functionally intact mitochondria from cultured cells and tissue biopsies. Anal Biochem 2013; 443(1):66–74.
